# Supplementary material for: ProteinShader: illustrative rendering of macromolecules
Source: BMC Struct Biol. 2009 Mar 30;9:19. doi: 10.1186/1472-6807-9-19 (PMC2672931; doi:10.1186/1472-6807-9-19)
Supplement: Additional file 1 — ProteinShader program without source code. This compressed file contains the complete ProteinShader program including associated libraries, but no source code. A README.txt file gives an overview of the ProteinShader distribution, and the index.html file in the help subdirectory has directions on getting started with the program as well as a set of tutorials. [file 1472-6807-9-19-S1.zip › ProteinShader-beta-0_9_4-binary/help/api/org/proteinshader/gui/MediatorImpl.html]

MediatorImpl (ProteinShader API)


|  |  |  |
| --- | --- | --- |
| **Overview** \| **Package** \| **Class** \| **Use** \| **Tree** \| **Deprecated** \| **Index** \| **Help** | |  |
| **PREV CLASS**   **NEXT CLASS** | **FRAMES**    **NO FRAMES**     **All Classes** |  |
| SUMMARY: NESTED \| FIELD \| CONSTR \| METHOD | DETAIL: FIELD \| CONSTR \| METHOD |  |


---


## org.proteinshader.gui Class MediatorImpl

```
java.lang.Object
  org.proteinshader.gui.MediatorImpl
```

**All Implemented Interfaces:**: Mediator

---

``` public class MediatorImpl extends Object implements Mediator ```

Serves as the central mediator central object that allows
communication between objects of the gui, the data structure, and
the renderer.

---

**Constructor Summary**

`MediatorImpl(ProteinShaderGUI gui,
GLCanvas canvas)`
  
          Constructs a ProteinShaderGUI.


| **Method Summary** | |
| --- | --- |
| `void` | `add(ControlPanel controlPanel)`             Adds the address of the ControlPanel to the Mediator. |
| `void` | `add(MainMenuBar mainMenuBar)`             Adds the address of the MainMenuBar to the Mediator. |
| `void` | `add(Renderer renderer)`             Adds the address of the Renderer to the Mediator. |
| `void` | `add(SpringLoadedJFrame controlFrame)`             Adds the address of the ControlFrame to the Mediator. |
| `void` | `blendObjectEdges(boolean b)`             Turns on or off the ability of the halftoning shaders to soften aliasing (jagged edges) by rendering translucent black silhouettes of tube and ribbon segments (and using jitter) before calling on the halftoning shaders. |
| `void` | `cacheGeometricObject(GeometricListInfo info)`             Caches a new geomtric object (sphere or cylinder) in the form of an OpenGL display list and redraws the canvas. |
| `void` | `clickStartAnimation()`             Calls on the startAnimation() method of the MotionPanel (equivalent to clicking the Start button on the MotionPanel). |
| `void` | `clickStopAnimation()`             Calls on the stopAnimation() method of the MotionPanel (equivalent to clicking the Stop button on the MotionPanel). |
| `void` | `closeControlFrame()`             Closes the spring-loaded frame holding the ControlPanel. |
| `void` | `displayErrorAndExit(String title, String message)`             Display an error message in a JOptionPane before exiting. |
| `void` | `displayWarning(String title, String message)`             Display a warning message in a JOptionPane. |
| `Vector<Texture>` | `getBendTextures()`             Returns the list of Textures intended for highlighting segment bend regions when halftoning is being used. |
| `Atom[]` | `getCurrentAtoms()`             Returns the current Atoms. |
| `Chain` | `getCurrentChain()`             Returns the current Chain. |
| `Model` | `getCurrentModel()`             Returns the current Model. |
| `Residue[]` | `getCurrentResidues()`             Returns the current Residues. |
| `CylinderListInfo` | `getCylinderInfo(StyleEnum style)`             Returns the CylinderListInfo object that holds the information on an OpenGL display list for a cylinder that is currently used for drawing Bonds. |
| `Frame` | `getFrame()`             Returns the frame that should be used as a parent frame for Dialogs. |
| `String` | `getGraphicsCardInfo()`             Returns information on the graphics card that was gathered when the init() method of the Renderer was called. |
| `Vector<Texture>` | `getHalftoningTextures()`             Returns the list of Texture objects intended for halftoning. |
| `Vector<Texture>` | `getPatternsTextures()`             Returns the list of Texture objects intended for placing patterns on colored surfaces (rather than textures for halftoning). |
| `SphereListInfo` | `getSphereInfo(StyleEnum style)`             Returns the SphereListInfo object that holds the information on an OpenGL display list for a sphere that is currently used for drawing Atoms. |
| `Structure` | `getStructure()`             Returns the current Structure to display. |
| `StyleEnum` | `getStyle()`             Returns the current style (TUBES, RIBBONS, RIBBONS\_AND\_TUBES, FRENET\_FRAMES, SPACE\_FILLING, BALLS\_AND\_STICKS, or STICKS). |
| `void` | `jitterScene(JitterEnum jitter)`             Draws the scene multiple times to an accumulation buffer while jittering the xy-coordinates a fraction of a pixel. |
| `void` | `openControlFrame()`             Opens the spring-loaded frame holding the ControlPanel. |
| `void` | `readStructure(File file)`             Reads in a new Structure to display. |
| `void` | `redrawCanvas()`             Redraws the canvas. |
| `void` | `setAutoTiling(boolean autoTiling)`             If automatic tiling is set to true, then the level of detail (tiling number) for a sphere or cylinder will be calculated based on the camera distance for each Atom or Bond, respectively. |
| `void` | `setBackgroundColor(float red, float green, float blue)`             Sets the background color for the canvas. |
| `void` | `setCurrentAtoms(Atom[] atoms)`             Sets the current Atoms. |
| `void` | `setCurrentChain(Chain chain)`             Sets the current Chain. |
| `void` | `setCurrentModel(Model model)`             Sets the current Model. |
| `void` | `setCurrentResidues(Residue[] residues)`             Sets the current Residues. |
| `void` | `setExtraLines(boolean b)`             Controls whether extra lines (at the beginning and end of each segment of a tube or ribbon) will be shown when the style is a cartoon type. |
| `void` | `setImageOrientation(OrientationEnum orientation)`             Sets the image to the requested orientation (Front, Back, Left, *etc*.). |
| `void` | `setImageScale(double scale)`             Sets the scale factor for the image. |
| `void` | `setRadioPanelSelectedMenu(String item)`             Sets the "Selected:" menu in the RadioPanel (for both atom and cartoon modifier panels) of the ModiferPanel. |
| `void` | `setStyle(StyleEnum style)`             Sets the current style (TUBES, RIBBONS, RIBBONS\_AND\_TUBES, FRENET\_FRAMES, SPACE\_FILLING, BALLS\_AND\_STICKS, or STICKS). |
| `void` | `showAminoAcids(boolean b)`             Controls whether the Renderer will display AminoAcids. |
| `void` | `showHeterogens(boolean b)`             Controls whether the Renderer will display Heterogens. |
| `void` | `showWaters(boolean b)`             Controls whether the Renderer will display Waters. |
| `void` | `startAnimation(double xAxisSpeed, double yAxisSpeed)`             Starts an animation that rotates the image about its x-axis and/or y-axis based on the speeds given as argument. |
| `void` | `stopAnimation()`             Stops the rotation that was started with the startAnimation() method. |
| `void` | `takeScreenShot(ImageFormatEnum format, File file)`             Causes the Render to take a screen shot of the current canvas image and save it as a JPEG, PNG or GIF file. |
| `void` | `updateFPSDisplay(double fps)`             During a rotation animation the Renderer will call this method to update the ControlPanel with the current frames per second the animation is operating at. |
| `void` | `updateRenderer()`             Updates the arrays of visible objects (opaque and translucent Drawables) held by the Renderer and then has the canvas call display(). |

**Methods inherited from class java.lang.Object**

`clone, equals, finalize, getClass, hashCode, notify, notifyAll, toString, wait, wait, wait`

**Constructor Detail**

### MediatorImpl

```
public MediatorImpl(ProteinShaderGUI gui,
                    GLCanvas canvas)
```

:   Constructs a ProteinShaderGUI.


**Method Detail**

### add

```
public void add(SpringLoadedJFrame controlFrame)
```

:   Adds the address of the ControlFrame to the Mediator.

    :   **Specified by:**: `add` in interface `Mediator`

---


### add

```
public void add(ControlPanel controlPanel)
```

:   Adds the address of the ControlPanel to the Mediator.

    :   **Specified by:**: `add` in interface `Mediator`

---


### add

```
public void add(MainMenuBar mainMenuBar)
```

:   Adds the address of the MainMenuBar to the Mediator.

    :   **Specified by:**: `add` in interface `Mediator`

---


### add

```
public void add(Renderer renderer)
```

:   Adds the address of the Renderer to the Mediator.

    :   **Specified by:**: `add` in interface `Mediator`

---


### getStructure

```
public Structure getStructure()
```

:   Returns the current Structure to display.

    :   **Specified by:**: `getStructure` in interface `Mediator`
    :   **Returns:**: The current Structure.

---


### readStructure

```
public void readStructure(File file)
```

:   Reads in a new Structure to display.

    :   **Specified by:**: `readStructure` in interface `Mediator`
    :   **Parameters:**: `file` - a PDB formatted file.

---


### redrawCanvas

```
public void redrawCanvas()
```

:   Redraws the canvas.

    :   **Specified by:**: `redrawCanvas` in interface `Mediator`

---


### getCurrentModel

```
public Model getCurrentModel()
```

:   Returns the current Model.

    :   **Specified by:**: `getCurrentModel` in interface `Mediator`
    :   **Returns:**: The current Model.

---


### getCurrentChain

```
public Chain getCurrentChain()
```

:   Returns the current Chain.

    :   **Specified by:**: `getCurrentChain` in interface `Mediator`
    :   **Returns:**: The current Chain.

---


### getCurrentResidues

```
public Residue[] getCurrentResidues()
```

:   Returns the current Residues.

    :   **Specified by:**: `getCurrentResidues` in interface `Mediator`
    :   **Returns:**: The current Residues as an array.

---


### getCurrentAtoms

```
public Atom[] getCurrentAtoms()
```

:   Returns the current Atoms.

    :   **Specified by:**: `getCurrentAtoms` in interface `Mediator`
    :   **Returns:**: The current Atom.

---


### getStyle

```
public StyleEnum getStyle()
```

:   Returns the current style (TUBES, RIBBONS, RIBBONS\_AND\_TUBES, FRENET\_FRAMES,
    SPACE\_FILLING, BALLS\_AND\_STICKS, or STICKS).

    :   **Specified by:**: `getStyle` in interface `Mediator`
    :   **Returns:**: The current style.

---


### getFrame

```
public Frame getFrame()
```

:   Returns the frame that should be used as a parent frame for
    Dialogs.

    :   **Specified by:**: `getFrame` in interface `Mediator`
    :   **Returns:**: The parent frame.

---


### cacheGeometricObject

```
public void cacheGeometricObject(GeometricListInfo info)
```

:   Caches a new geomtric object (sphere or cylinder) in the form of
    an OpenGL display list and redraws the canvas.

    :   **Specified by:**: `cacheGeometricObject` in interface `Mediator`
    :   **Parameters:**: `info` - describes the geometric object to cache.

---


### getSphereInfo

```
public SphereListInfo getSphereInfo(StyleEnum style)
```

:   Returns the SphereListInfo object that holds the information on an
    OpenGL display list for a sphere that is currently used for
    drawing Atoms. There are two types of spheres cached: one for
    SPACE\_FILLING and one for BALLS\_AND\_STICKS.

    :   **Specified by:**: `getSphereInfo` in interface `Mediator`
    :   **Returns:**: The display list info object for a sphere.

---


### getCylinderInfo

```
public CylinderListInfo getCylinderInfo(StyleEnum style)
```

:   Returns the CylinderListInfo object that holds the information on
    an OpenGL display list for a cylinder that is currently used for
    drawing Bonds. There are two types of cylinders cached: one for
    BALLS\_AND\_STICKS and one for STICKS.

    :   **Specified by:**: `getCylinderInfo` in interface `Mediator`
    :   **Returns:**: The display list info object for a cylinder.

---


### getPatternsTextures

```
public Vector<Texture> getPatternsTextures()
```

:   Returns the list of Texture objects intended for placing patterns
    on colored surfaces (rather than textures for halftoning).

---


### getHalftoningTextures

```
public Vector<Texture> getHalftoningTextures()
```

:   Returns the list of Texture objects intended for halftoning.

---


### getBendTextures

```
public Vector<Texture> getBendTextures()
```

:   Returns the list of Textures intended for highlighting segment
    bend regions when halftoning is being used.

---


### setCurrentModel

```
public void setCurrentModel(Model model)
```

:   Sets the current Model. The Renderer will be updated.

    :   **Specified by:**: `setCurrentModel` in interface `Mediator`
    :   **Parameters:**: `model` - the current Model.

---


### updateRenderer

```
public void updateRenderer()
```

:   Updates the arrays of visible objects (opaque and translucent
    Drawables) held by the Renderer and then has the canvas call display().

    :   **Specified by:**: `updateRenderer` in interface `Mediator`

---


### setCurrentChain

```
public void setCurrentChain(Chain chain)
```

:   Sets the current Chain.

    :   **Specified by:**: `setCurrentChain` in interface `Mediator`
    :   **Parameters:**: `chain` - the current Chain.

---


### setCurrentResidues

```
public void setCurrentResidues(Residue[] residues)
```

:   Sets the current Residues. The array will have a length of zero
    if no Residues are currently selected.

    :   **Specified by:**: `setCurrentResidues` in interface `Mediator`
    :   **Parameters:**: `residues` - the current Residues.

---


### setCurrentAtoms

```
public void setCurrentAtoms(Atom[] atoms)
```

:   Sets the current Atoms. The array will have a length of zero if
    no Atoms are currently selected.

    :   **Specified by:**: `setCurrentAtoms` in interface `Mediator`
    :   **Parameters:**: `atoms` - the current Atoms.

---


### setStyle

```
public void setStyle(StyleEnum style)
```

:   Sets the current style (TUBES, RIBBONS, RIBBONS\_AND\_TUBES, FRENET\_FRAMES,
    SPACE\_FILLING, BALLS\_AND\_STICKS, or STICKS).

    :   **Specified by:**: `setStyle` in interface `Mediator`
    :   **Parameters:**: `style` - the current style.

---


### setImageOrientation

```
public void setImageOrientation(OrientationEnum orientation)
```

:   Sets the image to the requested orientation (Front, Back, Left,
    *etc*.).

    :   **Specified by:**: `setImageOrientation` in interface `Mediator`
    :   **Parameters:**: `orientation` - the orientation to set the image to.

---


### setAutoTiling

```
public void setAutoTiling(boolean autoTiling)
```

:   If automatic tiling is set to true, then the level of detail
    (tiling number) for a sphere or cylinder will be calculated based
    on the camera distance for each Atom or Bond, respectively. If
    automatic tiling is set to false, then a standard sphere or
    cylinder will be used (and camera distance will not matter).

    :   **Specified by:**: `setAutoTiling` in interface `Mediator`
    :   **Parameters:**: `autoTiling` - boolean value for automatic tiling.

---


### showAminoAcids

```
public void showAminoAcids(boolean b)
```

:   Controls whether the Renderer will display AminoAcids.

    :   **Specified by:**: `showAminoAcids` in interface `Mediator`
    :   **Parameters:**: `b` - a boolean value indicating if AminoAcids should be displayed.

---


### showHeterogens

```
public void showHeterogens(boolean b)
```

:   Controls whether the Renderer will display Heterogens.

    :   **Specified by:**: `showHeterogens` in interface `Mediator`
    :   **Parameters:**: `b` - a boolean value indicating if Heterogens should be displayed.

---


### showWaters

```
public void showWaters(boolean b)
```

:   Controls whether the Renderer will display Waters.

    :   **Specified by:**: `showWaters` in interface `Mediator`
    :   **Parameters:**: `b` - a boolean value indicating if Waters should be displayed.

---


### setExtraLines

```
public void setExtraLines(boolean b)
```

:   Controls whether extra lines (at the beginning and end of each segment of a
    tube or ribbon) will be shown when the style is a cartoon type.

    :   **Specified by:**: `setExtraLines` in interface `Mediator`
    :   **Parameters:**: `b` - a boolean value indicating if extra lines should be used.

---


### openControlFrame

```
public void openControlFrame()
```

:   Opens the spring-loaded frame holding the ControlPanel. The
    "Tools" menu in the menu bar will be updated.

    :   **Specified by:**: `openControlFrame` in interface `Mediator`

---


### closeControlFrame

```
public void closeControlFrame()
```

:   Closes the spring-loaded frame holding the ControlPanel. The
    "Tools" menu in the menu bar will be updated.

    :   **Specified by:**: `closeControlFrame` in interface `Mediator`

---


### displayErrorAndExit

```
public void displayErrorAndExit(String title,
                                String message)
```

:   Display an error message in a JOptionPane before exiting.

---


### displayWarning

```
public void displayWarning(String title,
                           String message)
```

:   Display a warning message in a JOptionPane.

---


### startAnimation

```
public void startAnimation(double xAxisSpeed,
                           double yAxisSpeed)
```

:   Starts an animation that rotates the image about its x-axis and/or
    y-axis based on the speeds given as argument. An argument of zero
    results in no rotation, whild a negative argument results in
    clockwise instead of counterclockwise rotation.

---


### stopAnimation

```
public void stopAnimation()
```

:   Stops the rotation that was started with the startAnimation()
    method.

    :   **Specified by:**: `stopAnimation` in interface `Mediator`

---


### clickStartAnimation

```
public void clickStartAnimation()
```

:   Calls on the startAnimation() method of the MotionPanel (equivalent to
    clicking the Start button on the MotionPanel).

    :   **Specified by:**: `clickStartAnimation` in interface `Mediator`

---


### clickStopAnimation

```
public void clickStopAnimation()
```

:   Calls on the stopAnimation() method of the MotionPanel (equivalent to
    clicking the Stop button on the MotionPanel).

    :   **Specified by:**: `clickStopAnimation` in interface `Mediator`

---


### updateFPSDisplay

```
public void updateFPSDisplay(double fps)
```

:   During a rotation animation the Renderer will call this method to
    update the ControlPanel with the current frames per second the
    animation is operating at.

    :   **Specified by:**: `updateFPSDisplay` in interface `Mediator`
    :   **Parameters:**: `fps` - the frames per second for the animation.

---


### setImageScale

```
public void setImageScale(double scale)
```

:   Sets the scale factor for the image.

    :   **Specified by:**: `setImageScale` in interface `Mediator`
    :   **Parameters:**: `scale` - the scale factor as a double.

---


### setBackgroundColor

```
public void setBackgroundColor(float red,
                               float green,
                               float blue)
```

:   Sets the background color for the canvas.

    :   **Specified by:**: `setBackgroundColor` in interface `Mediator`
    :   **Parameters:**: `red` - the red component of the background color.: `green` - the green component of the background color.: `blue` - the blue component of the background color.

---


### takeScreenShot

```
public void takeScreenShot(ImageFormatEnum format,
                           File file)
```

:   Causes the Render to take a screen shot of the current canvas image and save
    it as a JPEG, PNG or GIF file.

    :   **Specified by:**: `takeScreenShot` in interface `Mediator`
    :   **Parameters:**: `format` - the screen shot image format.: `file` - the file to save the screen shot to.

---


### setRadioPanelSelectedMenu

```
public void setRadioPanelSelectedMenu(String item)
```

:   Sets the "Selected:" menu in the RadioPanel (for both atom and cartoon
    modifier panels) of the ModiferPanel.

---


### blendObjectEdges

```
public void blendObjectEdges(boolean b)
```

:   Turns on or off the ability of the halftoning shaders to soften aliasing
    (jagged edges) by rendering translucent black silhouettes of tube and
    ribbon segments (and using jitter) before calling on the halftoning shaders.
    This effect may be added to other shaders at a later time.

    :   **Specified by:**: `blendObjectEdges` in interface `Mediator`
    :   **Parameters:**: `b` - boolean value to determine if object edges should be smoothed.

---


### jitterScene

```
public void jitterScene(JitterEnum jitter)
```

:   Draws the scene multiple times to an accumulation buffer while jittering
    the xy-coordinates a fraction of a pixel.

---


### getGraphicsCardInfo

```
public String getGraphicsCardInfo()
```

:   Returns information on the graphics card that was gathered when the init()
    method of the Renderer was called.


---


|  |  |  |
| --- | --- | --- |
| **Overview** \| **Package** \| **Class** \| **Use** \| **Tree** \| **Deprecated** \| **Index** \| **Help** | |  |
| **PREV CLASS**   **NEXT CLASS** | **FRAMES**    **NO FRAMES**     **All Classes** |  |
| SUMMARY: NESTED \| FIELD \| CONSTR \| METHOD | DETAIL: FIELD \| CONSTR \| METHOD |  |


---

# *Copyright © 2007-2008*
